# Educating Enough Competent Health Professionals: Advancing Educational Innovation at Muhimbili University of Health and Allied Sciences, Tanzania

**DOI:** 10.1371/journal.pmed.1001284

**Published:** 2012-08-14

**Authors:** Ephata E. Kaaya, Sarah B. Macfarlane, Charles A. Mkony, Eligius F. Lyamuya, Helen Loeser, Phyllis Freeman, Edward K. Kirumira, Kisali Pallangyo, Haile T. Debas

**Affiliations:** 1 Muhimbili University of Health and Allied Sciences, Dar es Salaam, Tanzania; 2 University of California San Francisco, San Francisco, California, United States of America; 3 Journal of Public Health Policy, Boston, Massachusetts, United States of America; 4 Makerere University, Kampala, Uganda

## Abstract

Sarah MacFarlane and colleagues share their lessons engaging in educational reform and faculty development with the Muhimbili University of Health and Allied Sciences in Tanzania and the University of California San Francisco.

**Summary Points**

- To address Tanzania's extreme shortage of health professionals, increasing numbers of universities are training many more health professionals; for example, one university admitted about 50 medical students a year from 1971 until 1996, whereas in 2009 six universities admitted 756 medical students—still many fewer than are needed based on population growth.
- Universities can support health professionals to build and maintain critical competencies by strengthening curricula and pre-service and internship training, and providing opportunities for continuing professional development.
- Muhimbili University of Health and Allied Sciences (MUHAS), the oldest health sciences academic institution in Tanzania, is partnering with the University of California San Francisco to transform MUHAS's educational environment through curricula revision and faculty development.
- We learned that enhancing the educational process involves a great deal of commitment from faculty across MUHAS and will only succeed if supported by long-term institutional reform.
- Our work will take years to evaluate; sharing of early lessons learned by institutions undergoing educational reform will start to build a body of knowledge and experience to inform transformation of health professions education in Tanzania and elsewhere.

## The Need to Reform Health Professions Education in Tanzania

As immediate and crushing as the health burden is for Tanzania and across Africa, health systems will not cope better without many more health professionals competent to play leading roles in meeting population needs. Thus, national and international strategies depend fundamentally on universities, and on their transformation into modern engines for change and as custodians of quality education.

Tanzania suffers an extreme shortage of health workers [1]. A 2006 facility survey undertaken in Tanzania (Mainland and Zanzibar) suggests that about 39 nurses and midwives, between three and four medical doctors, and fewer than one pharmacist or dentist served a population of 100,000 [2]. The Ministry of Health and Social Welfare estimated that in 2006 about 29,000 government health staff served a Tanzanian population of approximately 38 million, 35% of the number needed [3].

Since independence, Tanzania has relied heavily on mid-level substitute health workers [4]. In 2006, 1,339 medical doctors, with five years training, comprised only 14% of Tanzania's medical workforce [2]. Clinical officers (CO), with three years training, in clinical medicine and community health, provide basic services in dispensaries (for 6,000 people); assistant medical officers (AMO), with two years clinical training beyond COs, provide clinical services in health centers (for 50,000) and in district hospitals (for 300,000). District and regional referral hospitals are meant to have physician supervisors, but COs and AMOs commonly work without such supervision, and thus beyond their levels of training.

To address the imperative for more health professionals, universities are expanding educational programs in Tanzania (Table 1), increasing intake of students [5], and introducing educational innovation [6],[7],[8]. To increase its teaching capacity, Muhimbili University of Health and Allied Sciences (MUHAS), for example, is expanding its teaching facilities by developing a new campus [9] and building its faculty. To ensure the quality of the services its graduates are able to provide, MUHAS is transforming its educational environment [10] in collaboration with educational institutions, ministries, professional bodies, and other stakeholders in Tanzania. MUHAS and the University of California San Francisco (UCSF) formed an academic partnership in 2005, and it has played a key role in curriculum innovation and faculty development [11],[12].

**Table 1:** Universities graduating health professionals in Tanzania.

University	Type of University, Location	Year of First Admissions	Undergraduate Educational Programs for Health Professionals
Muhimbili University of Health and Allied Sciences (MUHAS) (founded in 1963 as the School of Medicine, University College of Dar es Salaam, and chartered in 2007)	Public, Dar es Salaam	1963	Dentistry, environmental health, radiotherapy technology, laboratory sciences, medicine, nursing, pharmacy
International Medical and Technological University (IMTU)	Private, Dar es Salaam	1997	Laboratory sciences, medicine, nursing
Kilimanjaro Christian Medical College (KCMC), Tumaini University	Private, Moshi	1997	Medicine, nursing, physiotherapy
Hubert Kairuki Memorial University (HKMU)	Private, Dar es Salaam	1998	Medicine, nursing
Aga Khan University (AKU)	Private, Dar es Salaam	2001	Nursing
Catholic University of Health and Allied Sciences (CUHAS) (founded in 2003 as Bugando University College of Health Sciences, Saint Augustine University of Tanzania, and chartered in 2011)	Private, Mwanza	2003	Laboratory sciences, medicine, nursing, pharmacy
St John's University of Tanzania (SJUT)	Private, Dodoma	2007	Pharmacy, nursing
College of Health Sciences, University of Dodoma (UDOM)	Public, Dodoma	2009	Medicine, nursing

Source: authors (EEK, EL, and CM).

## Training and Loss of Graduate Health Professionals

Until 1997, MUHAS, then a college of the University of Dar es Salaam, was the sole institution training graduate health professionals in Tanzania. At MUHAS, medicine dates to 1963, pharmacy to 1974, public health (as community medicine) to 1976, dentistry to 1979, and nursing to 1988. In 2007, MUHAS gained its charter as a public university of health sciences and still teaches the largest number of health professional students. It is now joined by a new public university in Dodoma (admitting medical students from 2009) and seven private universities (1997–2010) (Table 1). The authors (EEK, EL, and CM) estimate that the national intake of medical students was stable at about 50 a year from 1971 to 1996, and quadrupled to 184 in 1999, and again to 756 in 2009; and that the annual intake in other health professions reached approximately 120 pharmacy (BPharm); 20 dental (DDS); 130 nursing (BScN); and 29 public health (BSc EHS).

The number of graduates who actually practice, however, disappoints: some never practice, while others gravitate to administrative jobs in non-governmental organizations or to work abroad. These losses of new entrants into health system positions result in part from 1) major delays in assigning graduates, during which time they seek employment elsewhere; and 2) the prospect of unattractive remuneration and benefits, and inadequate facilities that constrain the graduates' abilities to practice professionally once employed [13]. While it is the government's responsibility to redress these deficiencies, there are inadequacies in the education of health professionals that need to be addressed, in pre-service training (Box 1) and internships (Box 2), and there is a lack of opportunities for regulated and coordinated continuing professional development (Box 3).

**Box 1. Inadequacies in Pre-service Training of Health Professionals**

In 2009, to inform curriculum revision:

1. Teams of MUHAS faculty and students interviewed recent graduates in their workplaces, and their supervisors and co-workers;
2. Graduates from all the professional programs, and their supervisors, observed that newly employed graduates, although knowledgeable, lacked sufficient clinical or practical skills to meet workplace practice expectations;
3. Supervisors noted that graduates did not show sufficient professional respect for patients, colleagues, and the public;
4. Graduates recommended improving clinical training, more interactive learning, use of information technology, and stronger mentoring relationships with faculty.

**Box 2. Inadequacies in the Internship Experience**

1. All health professional graduates in Tanzania, wherever trained, are ministry of health employees through their internships, until obtaining licenses to practice;
2. Hospitals certified to meet the training requirements of the increasing numbers of graduates are too few, and apart from referral hospitals with a tradition of training interns, they are new to the task and have too few qualified supervisors;
3. Thus the training during internship rotations fails to meet educational objectives for many trainees.

**Box 3. Lack of a Formal System for Continuing Professional Development**

1. Tanzania lacks a formal system for coordinating continuing professional development;
2. Once licensed to practice by professional bodies, Tanzania does not require any health professional in Tanzania to update or improve skills or knowledge;
3. This negatively impacts professional standards, motivation of health workers, and ultimately health outcomes.

## Transforming MUHAS's Educational Environment

In 2008, concerned about the quality of tertiary education, the government of Tanzania called on all universities to introduce competency-based education. MUHAS embraced this mandate as an opportunity to initiate a planned transformation of its educational environment. The vision features MUHAS as a center of excellence to catalyze reform of health professions education throughout Tanzania.

Core activities we describe derive from the MUHAS-UCSF Academic Learning Project [11], one of a group of projects supported by the Bill & Melinda Gates Foundation [14]. UCSF is the University of California's only campus dedicated exclusively to health sciences, with schools of dentistry, medicine, nursing, and pharmacy, and a graduate division. UCSF's recent experience in curricular transformation in its own health professions schools [15],[16],[17], along with its commitment to global health [18], proved particularly relevant in facilitating MUHAS's process of innovation and change.

### Building Faculty Leadership

Few MUHAS faculty had any formal training as educators and most were new to competency-based education. In 2010, approximately 40% of 217 faculty members in MUHAS's five professional schools were about five years from retirement or already post-retirement, working on contract. MUHAS decided to invest in young faculty as future academic leaders, engaging their enthusiasm and energy to create a contemporary and supportive educational environment.

MUHAS leadership recruited an inter-professional team of mostly junior faculty who later gained recognition and authority as the Health Professions Educators Group (HPEG). Working alongside UCSF educators in a series of intensive workshops, the group developed expertise and prepared training sessions on teaching and assessment methods required for competency-based education. These activities regularly expand MUHAS faculty and postgraduate student engagement with educational improvement for better health outcomes.

### Preparing Health Professionals

MUHAS established curriculum committees at university, school, institute, and departmental levels. The committees reviewed “competency domains" used in health professions schools in the United States (US), Australia, and the United Kingdom, assessed their relevance in Tanzania, and identified eight for MUHAS graduates, each associated with two to ten competencies. In six domains, the competencies are identical for all professional programs; these domains are: relationships with patients, clients and communities; relationships with colleagues; teaching skills; maintaining good practice; working within the system and context of health care; and professionalism. Within the domains of professional knowledge and practical/clinical skills the competencies are profession-specific. MUHAS designed its competencies to improve health outcomes; the new framework for training of health professionals from all schools is geared to improving performance of graduates, including supporting their participation in inter-professional practice.

Advised by ministries responsible for education and health, education experts from other Tanzanian universities, and professional bodies—and informed by their 2009 survey of graduates—MUHAS faculty constructed the new curricula. With support from the committees and the HPEG, the faculty designed methods of instruction (including team-based learning, clinical simulations, community-based learning, and use of information technology) and assessment of student achievement of competencies (including objective structured clinical examinations, multisource assessment. and structured multiple choice questions). The Tanzanian Commission for Universities, the national accreditation body, approved the final curricula in September 2011; the MUHAS launch followed in October 2011 for all matriculating students.

### Updating Policies and Facilities

To support and maintain the momentum around education sparked by the introduction of competency-based education, MUHAS leadership introduced policies and procedures that 1) updated merit-based promotion criteria to emphasize faculty contributions to teaching and educational scholarship; 2) highlighted educational scholarship as a research theme, with associated small grants; 3) incorporated the HPEG workshops into a formal faculty development program; and 4) mandated an educational course for all postgraduate trainees to integrate teaching and mentoring into their careers, some as future faculty.

MUHAS plans to open a Centre for Health Professions Education to provide a physical environment to stimulate more inter-disciplinary educational innovation attuned to inter-professional teamwork needs. State-of–the-art teaching, learning, and assessment facilities will serve faculty and students at MUHAS and other Tanzanian universities and promote continuing professional development among practitioners nationwide.

## Lessons Learned

The work we describe took several years of relationship building, needs assessments, and piloting to prepare, and three intense years of collaboration, skill building, and technological upgrades to carry out; but it will require a decade or more to fully implement and evaluate. In 2010, the *Lancet* Commission on Health Professions Education for the 21^st^ Century urged rethinking of health professions education worldwide, focusing on the competencies required to meet evolving health priorities. The Commission argued that if health and education systems work together, collaborating across professions and sectors, there will be greater impact on population health outcomes [19]. Taking advantage of our relatively early start in such a process, we highlight in Boxes 4 and 5 the lessons learned so far, and their importance for carrying MUHAS's learning into field settings to support young graduates to practice.

**Box 4. Lesson: Enhance the Educational Environment through Institutional Reform**

MUHAS learned it needed new approaches and policies to effect and sustain its desired transformation. Chief among these are changes to:

1. Prepare, retain, and sustain faculty as teachers, and recognize their contributions to teaching and educational scholarship;
2. Expand teaching resources by grooming postgraduates as potential faculty and training practicing professionals as adjunct faculty;
3. Encourage pilots to inform mainstreaming of competency-based curricula attuned to health needs, and to update teaching and student assessment;
4. Promote a constant process of evaluating all aspects of change in order to inform next steps.

**Box 5. Lesson: Advance Change from Competency-Based Education to Workplace Proficiency Country Wide**

To assure the desired workforce transformation, Tanzania's introduction of competency-based education for the health professions will require clear continuity beyond MUHAS and other professional schools—moving into professional settings, including designation of responsibility for supervision, oversight, and accreditation beyond the initial degree.

In Tanzania, as in other African countries, graduation concludes the university role; the ministry of health supervises internships. Universities, ministries of education and health, and accreditation, regulatory, and professional bodies will need to work together in the formation and nurturing of competence. They will also need to share responsibility for supervising and accrediting the achievement of improving competence through the professional continuum. In opening its Centre for Health Professions Education, MUHAS plans to coordinate players to address some of these issues on a national scale [11].

**Box 6. Lesson: Leverage Collaboration to Create Centers of Academic Excellence and Educational Innovation**

Promoting appropriate curricula and progressive educational strategies and methods is essential for transformative scale-up of health professions education for better health outcomes. MUHAS is demonstrating the role of a university as an innovator, “incubator", and disseminator of know-how for advancing education—with the benefit of its UCSF partnership. We learned that health professional schools need to:

1. Share resources and team up across professional schools, then collaborate among health universities across the country and across professions, sharing lessons about improving education and managing change;
2. Strive for inter-professional educational environments and training that allow students to learn, and subsequently work—together in inter-professional teams;
3. Invite strategic international partnerships with collaborators willing and able to support innovation consistent with national strategies.

The future of MUHAS's effort to enhance its educational environment lies in its faculty. As in other African universities [20], shortages loom as senior faculty rapidly retire. While building the capacities of younger faculty, and retaining experienced faculty after retirement at 60 years of age, MUHAS is actively developing and recruiting new faculty. Working with the Public Service Commission, MUHAS is negotiating for more faculty positions to fill through recruitment from its specialized master's programs. MUHAS intends to attract masters students to become faculty through 1) a mandated course in teaching as preparation to become faculty; 2) opportunities to obtain PhDs through sandwich arrangements with partner universities (for example with the Karolinska Institute and Umeå University in Sweden, and the University of Bergen in Norway); and 3) opportunities to train with partner institutions through research funding (for example, with the universities of Dartmouth and Harvard in the US, Kwa Zulu Natal in South Africa, Maastrich in the Netherlands, Uppsala in Sweden, and the Vellore Christian Medical College in India). Partner support for faculty and administrators will be essential over the short-term.

Just as MUHAS is transforming its curricula and developing faculty in partnership with UCSF, other newer universities in Tanzania are introducing educational innovations with partner support. The Kilimanjaro Christian Medical College is working with Duke University School of Medicine to review and revise its curricula and to introduce problem-based learning with support from the US National Institutes of Health Fogarty Medical Educational Partnership Initiative [6],[21]; and the Catholic University of Health and Allied Sciences has long been working with the TOUCH Foundation [7] and the Weill Cornell Medical School to expand student intake by enhancing facilities and teaching capacity. Given the lack of information about educational developments in Africa [22] and the time it takes to accumulate evidence in education, we believe it important for universities to share lessons learned from work-in-progress, and to demonstrate that the *Lancet* Commission's proposals [19] are feasible.

## Abbreviations

AMO: assistant medical officers
CO: clinical officers
HPEG: Health Professions Educators Group
MUHAS: Muhimbili University of Health and Allied Sciences
UCSF: University of California San Francisco
